# Abscessed Uterine and Extrauterine Adenomyomas with Uterus-Like Features in a 56-Year-Old Woman

**DOI:** 10.1155/2013/238156

**Published:** 2013-07-17

**Authors:** Asiye Safak Bulut, Tevfik Uğur Sipahi

**Affiliations:** ^1^TOBB ETU Hospital, Department of Pathology, Yasam cad. No:5 Sogutozu, 06510 Ankara, Turkey; ^2^TOBB ETU Hospital, Department of Gynaecology, Ankara, Turkey

## Abstract

Adenomyosis, also known as endometriosis interna, is the presence of endometrial glands and stroma within the myometrium. Its localised form is called adenomyoma and mimics a leiomyoma. Rarely, adenomyomas are located outside the uterus and some of them form uterus-like masses with a thick muscle wall and an endometrium-lined central cavity. They are generally located in the ovary or broad ligament, and, although they are closely related to endometriosis, their pathogenetic mechanisms are different from each other. Müllerian duct fusion defect and subcoelomic mesenchyme transformation theory are two possible pathogenetic mechanisms for this rare entity. Here we report abscessed uterine and extrauterine adenomyomas forming uterus-like masses in the left and right broad ligament and an ectopic adrenal tissue in the left paraovarian region in a 56-year-old woman. Although there is a reported abscessed adenomyosis in the literature, this is the first abscessed extrauterine uterus-like masses with synchronous pelvic pathologies like endometriosis, leiomyomas, adenomyosis, an endometrial polyp, an endocervical polyp, and an ectopic adrenal tissue. This benign lesion gives the impression of leiomyoma, a uterine malformation, or even malignancy preoperatively. Frozen section helps in differential diagnosis.

## 1. Introduction

An adenomyoma is a circumscribed, nodular aggregate of smooth muscle, endometrial glands, and, usually, endometrial stroma within the myometrium [1]. The downgrowth and invagination of the basalis endometrium into the myometrium are the current developmental theory [2]. Although there are few reports describing abscess formation in endometriosis, there is only one reported case of abscessed adenomyosis [3–5]. Adenomyomas may be located within the myometrium, or it may involve or originate in the endometrium and grow as a polyp [1, 6]. Extrauterine adenomyomas located at the ovary, broad ligament, and uterosacral ligament are rare, and those mimicking a uterus are even rarer [7–11]. Here we report multiple abscessed adenomyomas forming uterus-like masses in the left and right broad ligament and in myometrium. There were also leiomyomas and adenomyosis in the myometrium, adhesions in the pelvis, and fluid in Douglas fossa. The diagnosis was made by frozen section. Postoperative period was uneventful.

## 2. Case Presentation

**Figure 1:**
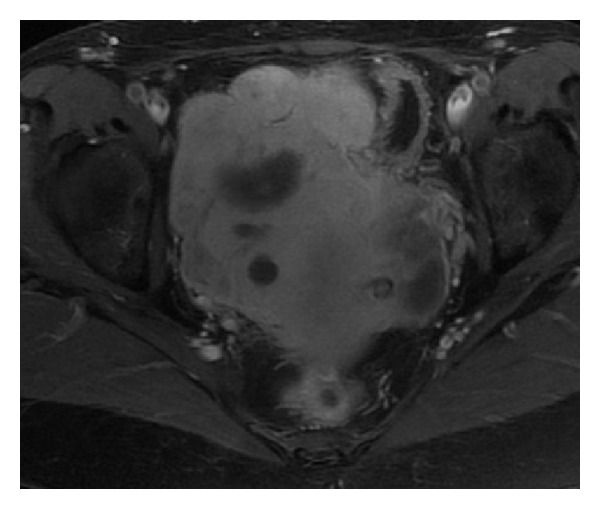
Lobulated mass, filling the pelvis and indenting uterus in MRI.

A 56-year-old multiparous (gravida 2, para 2) woman presented with menorrhagia after three months' amenorrhea and pelvic and lumbar pain. There was no history of a chronic disease, pelvic inflammatory disease, intrauterine device, or tubal pathology. On physical examination, she was found to have lobulated hard masses filling the pelvis up to the umbilicus. She had no fever. Transvaginal ultrasonography showed well-defined nodular masses around the uterus obscuring the discrimination between uterus and ovaries. Serum CA 19-9 and CA-125 levels were 32.94 U/mL and and 46.60 U/mL, respectively. In the whole blood, erythrocyte, leukocytes, and thrombocyte counts were 3.11 × 10^6^/UL, 16.64 × 10^6^/UL, and 165 × 10^6^/UL, respectively. Hemoglobin was 9.8 g/dL; haematocrit was 28.5%. CRP was 249.1 mg/L. Urine culture was negative. Magnetic resonance imaging revealed a giant hypervascular lobulated mass measuring 8 × 12 × 13 cm, with central necrosis, filling the pelvis, indenting the uterus, rectosigmoid, and bladder. It was closely related to uterine corpus so it was thought to be originated from it (Figure 1). It was reported to be a large necrotic leiomyoma without an exclusion of malignancy. Laparotomy was planned in order to rule out a pelvic malignancy.

Intraoperative evaluation revealed four leiomyoma-like masses in the right broad ligament measuring 5 to 10 cm in diameters. There was a 6-cm-diametered one in the left broad ligament and two other ones measuring 7 cm and 5 cm in the posterior and right walls of the uterus. There were also intraperitoneal adhesions and 20 cc fluid in the Douglas fossa. One of the masses located in the right broad ligament was sent for frozen evaluation. It was a peritonealised surfaced nodular mass with 8 × 7 × 5 cm dimensions and had a 4 × 1 cm central cavity containing pus. The cut surface of the mass was somewhat like a uterus with a thick muscle wall and an endometrial cavity-like central space with some nodularity in the wall. 

**Figure 2:**
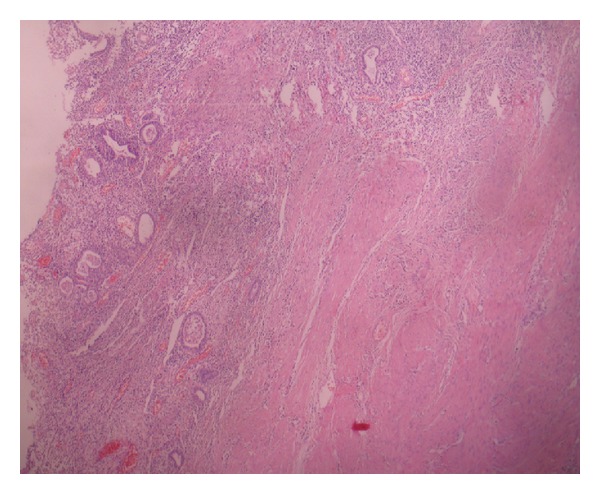
Inflamed endometrium-lined inner surface of uterus-like mass (HE, original magnification is ×40).

In microscopic examination, the cavity was lined by endometrial type glands and stroma and infiltrated by leukocytes, lymphocytes, plasmocytes, hemosiderin-laden macrophages, and hystiocytes (Figure 2). The inflammation was extending to the thick muscle wall. There were small endometriotic foci and small leiomyomas between the muscle bundles. There was no architectural complexity or cytologic atypia within the epithelium, and mitotic figures were infrequent. The smooth muscle component was arranged in irregular fascicles. There was no mitotic activity or necrosis. As the mass was reminiscent of a uterus macroscopically and microscopically, it was thought to be one of the corns of a bicornuate uterus. But it was learned that it was not connected to the uterine body, tube, or ovary. There were also other similar masses in the pelvis. So a diagnosis of abscessed adenomyoma was made with frozen section. Total abdominal hysterectomy and bilateral salpingo-oophorectomy with excision of intraligamentary masses and intraperitoneal adhesions were performed. 

In the hysterectomy specimen, there was a similar mass with 9.5 cm diameter in the right wall of the uterus. There were Nabothian cysts and a 15 × 10 × 4 mm polypoid lesion in the cervix and a 4 × 3 × 2 mm polyp in the endometrial cavity. Five leiomyomas up to 25 mm diameter and a few small bloody cysts were observed within the myometrium. The left wall of the uterus was yellowish in color. Ovaries contained small cysts, and there was a 4 mm orange colored nodule between the left ovary and left tuba. 

**Figure 3:**
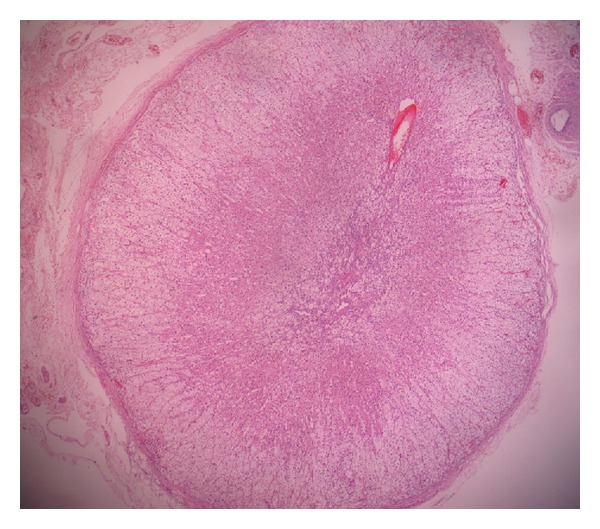
Ectopic adrenal tissue (HE, original magnification is ×40).

The right-sided uterine mass and intraligamentary masses had a similar microscopic feature with the one evaluated by frozen section. There were endometriotic foci and leiomyomas, one of which is palisaded type, in the myometrium. Ovaries contained follicular cysts. The orange colored nodule was consistent with an ectopic adrenal tissue mostly formed of adrenal cortical cells (Figure 3). 

In the microbiological examination, no microorganisms were isolated from the foci of abscess in both aerobic and anaerobic cultures, and there was not another primary source of infection. So, the cause of inflammation remained unknown. The patient's postoperative period was uneventful. 

## 3. Discussion

The presence of tumor-like masses formed of endometrial glands, specialized endometrial stroma, and smooth muscle in extrauterine localisations is rare, and the terminology is somewhat arbitrary for these lesions. The term “extrauterine adenomyoma” is preferred by most authors as they are similar in most respects to their more common uterine counterparts [7, 8, 12]. Some other authors used the term “uterus-like mass” as an alternative terminology [9, 10, 13–15]. Adenomyomas that share some features of uterus-like masses are described as “adenomyoma with uterus-like features” by some authors [16–18]. Matsuzaki et al. used the term “endomyometriozis” for a mass lesion in the uterosacral ligament [11]. Our case had the features of both adenomyoma and uterus-like masses and defined as “adenomyomas with uterus-like features”.

Although the first uterus-like mass is said to be described by Cozzutto in 1981, Cranstoun reported a cystic adenomyoma of uterus in 1922 [13, 19]. The mass he presented was very dark colored and adherent to the parietal peritoneum, omentum, and gut by its anterior surface. The rupture of the mass caused the escape of large quantities of brown muddy-looking fluid. Uterus was irregularly enlarged and contained three fibromyoma-like masses. Each mass was composed of uterine tissue containing many spaces filled with degenerated blood-clot and communicated with the others. The large cyst was continuous with the edge of the largest fibromyoma-like mass that was located at the upper left corner of the uterus. The cystic mass was also composed of uterine tissue with many spaces filled with degenerated blood-clot in the wall. Left and right ovaries and tubas were macroscopically normal. The explanation of the mass was the degeneration and distention that started centrally and expended to the upper part of the tumor that least resistance was encountered on the abdominal site. Although the diagnosis was a cystic adenomyoma of the uterus, their uterine tissue composition and the presence of endometrium-like cavity and blood-clot in the center were so reminiscent of uterus-like masses later defined.

Carinelli et al. stated that there are only 19 extrauterine adenomyomas in the English-language scientific literature [18]. These uterus-like masses can be located in various extrauterine sides including the broad ligament, ovary, and uterosacral ligament [11, 14, 16, 17]. They typically exhibit an organoid arrangement consisting of a single central cavity lined by endometrial type mucosa with surrounding myometrial like tissue. Our case had such an organoid arrangement with small endometriotic foci and leiomyoma-like arrangements in the smooth muscle component. The uterine and extrauterine adenomyomas were abscessed in our case. Although abscess formation is reported in the endometriomas [3, 4], only Erguvan et al. reported an abscessed adenomyosis [5]. In Martino et al.'s case the abscess occurred iatrogenically following fine needle aspiration [3]. Lipscomb et al. hypothesized that the abscess in their case might have developed following haematogenous spread of bacteria from an urinary tract infection because the same organism was recovered from the patient's urine and the abscess cavity [4]. In Erguvan et al.'s case, no microorganisms were isolated from the foci of abscess in both aerobic and anaerobic cultures, and the cause of infection remained unknown as in our case. 

A Müllerian duct fusion defect is the possible mechanism for the pathogenesis of uterus-like masses. This theory is based on a developmental abnormality occurring during the formation of the female genital tract. Lack of fusion of the Müllerian ducts in a localized area, or throughout the length of the ducts, may explain various duplications or atresias of the uterus [20]. In our case, the patient has a normal uterus, cervix, fallopian tubes, and ovaries. She had no renal abnormality.

Another theory is the subcoelomic mesenchyme transformation theory [9, 18]. The subcoelomic mesenchyme or secondary Müllerian system is defined as the layer of tissue that lies underneath the mesothelial surface of the peritoneum. The proliferation of this mesenchyme may give rise to mesenchymal lesions composed of endometrial stromal-type cells, desidua, or smooth muscle. 

Batt et al. suggested the theory of müllerianosis—developmentally misplaced Müllerian tissue—for their case [21]. In 2007, they defined müllerianosis by clinical-pathologic criteria as a heterotopic organoid structure of embryonic origin, a choristoma composed of Müllerian rests—normal endometrium, normal endosalpingeal tissue, and normal endocervical tissue—incorporated singly or in combination within other normal organs during organogenesis. He stated that Müllerian choristomas with only one Müllerian tissue, such as parauterine uterus-like mass, can be diagnosed with a high degree of probability only when three criteria are met: (1) no evidence of pelvic endometriosis, (2) no direct communication with the endocervix, endometrium, or endosalpinx, and (3) no history of surgery on the reproductive organs. 

The differential diagnosis includes endometrioma with a smooth muscle component, leiomyomatosis peritonealis disseminata, extrauterine leiomyoma with entrapped endometrioid glands and stroma (adenomyotic leiomyoma), [22–24], uterine malformation [9], and malignancy. The thick smooth muscle component distinguishes the mass from endometrioma and adenomyotic leiomyoma. The presence of cycling endometrium excludes leiomyomatosis peritonealis disseminata. Lack of atypia, mitosis, and necrosis in endometrial and smooth muscle component excludes malignancy.

Surgical treatment followed by long-term GnRH agonist therapy is said to be effective in keeping the disease stable [18]. As there are two reported extrauterine adenomyomas with features of uterus-like masses that occurred 17 and 22 years after hysterectomy, a long term followup is essential for these patients [16, 17]. Treatment with broad-based antibiotics can be unsuccessful for the abscessed ones.

The presence of ectopic adrenal tissue in the adnexal region is an interesting finding in our case. Although it was incidentally found, the development of this ectopic gland may be related to the development of uterus-like masses embryologically because the adrenal cortex develops from the coelomic mesodermal epithelium, that is closely related to Müllerian system, in the fourth to sixth week of gestation as a cluster of cells between the root of mesentery and the genital ridge [25]. Ectopic adrenal tissue can migrate with gonadal tissues. Therefore, adrenal rests can be found anywhere along the path of embryonic migration, including kidney, periadrenal, and retroperitoneal fat, in proximity to pelvic organs such as ovary, uterus, broad ligament, spermatic cord and vessels, and testes [26]. It is generally accepted that adrenal rests are due to mechanical separation and displacement of portions of cortical tissue during migration and descent of the sex glands. As coelomic epithelium is immediately adjacent to the developing Müllerian duct, a Müllerian duct fusion defect can cause mechanical separation of adrenal tissue.

In Schechter's report, Morgagni was said to be first described yellowish nodules resembling adrenal tissue adjacent to the main glands in 1740 [27]. Accessory glands are usually composed of cortical tissue only, and they are said to occur in 50% of newborns. They regress and disappear within a few years. In adults, they are generally found incidentally, in association with pelvic structures, such as the broad ligament, spermatic and ovarian vessels, spermatic cord, ovary, canal of Nuck, and uterus [26, 28].

The clinical implication, of the adrenal rests are essential in the surgical approach of the patients. In patients who have undergone bilateral adrenalectomy due to pathologic ACTH production, compensatory hyperplasia of the ectopic adrenal tissue may be responsible for the recurrence of the disease. Another clinical aspect is the possibility of formation and development of malignant disease in the ectopic gland. 

Here we reported an interesting case with uterine and extrauterine abscessed adenomyomas with features of uterus-like masses. There was an ectopic adrenal tissue in the adnexal region. Although the ectopic tissue seems to be incidentally found, the development of these two separate entities may be related to each other embryologically.
